# The Influence of L-Lysine-Alpha-Oxidase on the Biofilm Formation of Opportunistic Microorganisms Associated with Inflammatory Diseases of the Urinary Tract

**DOI:** 10.3390/pathogens13030252

**Published:** 2024-03-15

**Authors:** Alexandr Senyagin, Nadezhda Sachivkina, Milana Das, Anna Arsenyuk, Ramziya Mannapova, Alfir Mannapov, Tursumbai Kubatbekov, Dmitriy Svistunov, Olesya Petrukhina, Andrey Zharov, Natallia Zhabo

**Affiliations:** 1 Department of Microbiology V.S. Kiktenko, Medical Institute, RUDN University Named after Patrice Lumumba, 117198 Moscow, Russia; senyagin_an@pfur.ru (A.S.); krikun_ms@pfur.ru (M.D.); 2 All-Russian Research Institute for Veterinary Sanitation, Hygiene and Ecology—Branch of Federal Scientific Center—K.I. Skryabin, Ya.R. Kovalenko All-Russian Research Institute of Experimental Veterinary Medicine, Russian Academy of Sciences, 109428 Moscow, Russia; aarsenuk@gmail.com; 3 Department of General Pathology, Moscow State Academy of Veterinary Medicine and Biotechnology named after K. I. Skryabin, 109472 Moscow, Russia; 4 Department of Veterinary Medicine, Russian State Agrarian University, Moscow Timiryazev Agricultural Academy, 127434 Moscow, Russia; ram.mannapova55@mail.ru (R.M.); 54alfir@mail.ru (A.M.); tursumbai61@list.ru (T.K.); dimitriisvist@mail.ru (D.S.); 5 Department of Veterinary Medicine, Agrarian Technological Institute, RUDN University Named after Patrice Lumumba, 117198 Moscow, Russia; petrukhina-oa@rudn.ru; 6 Department of Technosphere Security, Agrarian Technological Institute, RUDN University Named after Patrice Lumumba, 117198 Moscow, Russia; zharov-an@rudn.ru; 7 Department of Foreign Languages, Medical Institute, RUDN University Named after Patrice Lumumba, 119034 Moscow, Russia; lys11@yandex.ru

**Keywords:** biofilm formation, *Escherichia coli*, *Candida* spp., *Pseudomonas aeruginosa*, *Proteus mirabilis*, *Staphylococcus aureus*, L-lysine-α-oxidase, *Trichoderma harzianum*, catheter-associated urinary tract infection

## Abstract

Urinary tract infections occupy a special niche among diseases of infectious etiology. Many microorganisms associated with urinary tract infections, such as *Klebsiella oxytoca*, *Enterococcus* spp., *Morganella morganii*, *Moraxella catarrhalis*, *Pseudomonas aeruginosa*, *Proteus mirabilis*, *Staphylococcus aureus*, *Staphylococcus* spp., and *Candida* spp., can form biofilms. The aim of this research was to study the effect of the enzyme L-lysine-Alpha-oxidase (LO) produced by the fungus *Trichoderma harzianum Rifai* on the biofilm formation process of microorganisms associated with urinary tract infections. Homogeneous LO showed a more pronounced effect than the culture liquid concentrate (cCL). When adding samples at the beginning of incubation, the maximum inhibition was observed in relation to *Enterococcus faecalis* 5960—cCL 86%, LO 95%; *Enterococcus avium* 1669—cCL 85%, LO 94%; *Enterococcus cloacae* 6392—cCL 83%, LO—98%; and *Pseudomonas aeruginosa* 3057—cCL 70%, LO—82%. The minimum inhibition was found in *Candida* spp. Scanning electron microscopy was carried out, and numerous morphological and structural changes were observed in the cells after culturing the bacterial cultures in a medium supplemented with homogeneous LO. For example, abnormal division was detected, manifesting as the appearance of joints in places where the bacteria diverge. Based on the results of this work, we can draw conclusions about the possibility of inhibiting microbial biofilm formation with the use of LO; especially significant inhibition was achieved when the enzyme was added at the beginning of incubation. Thus, LO can be a promising drug candidate for the treatment or prevention of infections associated with biofilm formation.

## 1. Introduction

The leading etiological agent in terms of bacterial urinary tract infections is uropathogenic *Escherichia coli* (UPEC). UPEC possesses an extensive set of virulence factors, such as hemolysins, R-plasmids, beta-lactamases, and QS proteins [1]. These determine its ability to develop antibiotic resistance and form biofilms; these properties have been proven to correlate with each other [2]. In a few clinical cases, this makes the complete eradication of the pathogen extremely difficult [1,2].

However, in addition to UPEC, there are other microorganisms associated with urinary tract infections. Among the uropathogenic group of microorganisms, there are the following: *Klebsiella oxytoca*, *Enterococcus* spp., *Morganella morganii*, *Moraxella catarrhalis*, *Pseudomonas aeruginosa*, *Proteus mirabilis*, *Staphylococcus aureus*, *Staphylococcus* spp., *Candida* spp., etc. It is important to note that, according to the literature, almost all of the above pathogens are capable of biofilm formation [3,4,5,6,7,8,9], which certainly leads to complications in the treatment of urinary tract infections.

The listed pathogens are extremely active in nosocomial infections, infections in immunosuppressed patients, mixed infections, and secondary infections, and during the postoperative period, they can contribute to iatrogenic complications, a special place among which are complications from urinary catheterization [6,10]. This manipulation is widely used, but when catheterizing the bladder for more than 2 days, it is customary to talk about the potential development of catheter-associated urinary tract infection (CAUTI), which has a 90% probability [11]. The entry of a foreign body into the lumen of the urethra or bladder greatly increases the risk of the formation of biofilms by microorganisms, both on the foreign body itself and on the urinary tract epithelium due to the trauma caused and the release of cellular elements, prothrombin, and other elements that act as mediators in the process of adhesion and coaggregation. All of the above makes it relevant to study the formation of biofilms and the inhibition of this process comprehensively.

Urinary tract infections occupy a special niche among diseases of infectious etiology, since many clinical cases, up to 80%, become chronic [12,13,14]. This is due to a number of factors: anatomical features (the proximity of anatomical openings, which promotes the migration of opportunistic microorganisms from the intestine), histological factors (features of the transitional epithelium of the urethra and bladder—the ability to form intercellular spaces during the inflammatory process—where microorganisms easily multiply), and characteristics of the pathogens themselves, for example, their adhesion abilities and subsequent biofilm formation [12,13,14]. The ability of pathogenic and non-pathogenic microorganisms to form biofilms is one of the leading problems in the field of therapy and prevention of infectious diseases [15]. Formed biofilms prevent the penetration of drugs into bacteria and, as a result, complicate or prevent the antibacterial eradication of the pathogen, which in turn leads to the difficulty and prolongation of treatment, a chronic course of the inflammatory process, and subsequent relapses [15,16].

There are different approaches to treating inflammation associated with biofilm formation. The use of natural or synthetic compounds that affect virulence factors and quorum sensing (QS) is one of the most promising areas of research [6]. Among alternative strategies for antibiotic therapy, the use of microbial adhesion inhibitors stands out, the most famous of which is a chelator that competitively binds Fe^2+^, Zn^+^, and magnesium ions, which are critical for biofilm formation [8]. Other methods include the carrier-mediated diffusion of antimicrobial drugs into the biofilm matrix and bacteriophage therapy, which has demonstrated successful results in vitro [6,17,18]. Another promising direction is the use of enzyme-based drugs [18], which will influence the process of biofilm formation and/or the destruction of an already formed one.

Theoretically, a promising enzyme could be L-lysine-α-oxidase (LO), an enzyme belonging to the class of oxidoreductases of the amino acid L-lysine. The enzyme is an oxidoreductase of the amino acid L-lysine, and it was first studied by the Japanese researcher H. Kusakabe [19]. Imperfect fungi of the genus *Trichoderma* produce LO. A few studies have shown its antitumor [20,21] and antifungal [15,22] activities. Previously, our study demonstrated the effect of LO produced by the fungus *Trichoderma harzianum Rifai* on the process of biofilm formation in UPEC [23].

The aim of this work was to study the effect of the enzyme L-lysine-α-oxidase produced by the fungus *Trichoderma harzianum Rifai* on the process of biofilm formation of microorganisms associated with urinary tract infections.

## 2. Materials and Methods

### 2.1. Cultivation of the Producer Trichoderma harzianum Rifai and Production of the Enzyme

The inoculum of the producer *Trichoderma harzianum Rifai* was pre-grown on Sabouraud medium (HiMedia, Mumbai, India) for 14 days at a temperature of 27 ± 1 °C until it fully morphologically corresponded to the mature culture. After preparing the inoculum, it was cultured on a medium of Soda (500 mL of distilled water, 200 g of wheat bran, 8 g of NaNO_3_) in a shaker/incubator (Heidolph Unimax 1010, Heidolph, Schwabach, Germany) at a temperature of 27 ± 1 °C at 80–100 rpm for the constant aeration of the medium and continuous mixing of the substrate. The culture was grown under the following conditions: temperature: 28 ± 1 °C; pH of the environment: 5.8–6.0. If the pH increased above 7.0, a solution of 10% hydrochloric acid was added to the flask. The process was controlled by the pH of the medium. The level of LO activity was visualized using PAGE (polyacrylamide gel electrophoresis). Control samples were taken every 24 h. The total fermentation time took 288 h.

Upon the completion of fermentation of the producer in the medium of Soda, the culture liquid was sent for preliminary purification. The fungal mycelium and large particles of the medium were separated from the liquid part in ultrafiltration cells using the following membranes: Membrane Filter, 0.22 μm (D = 150 mm, MERCK Millipore, Darmstadt, Germany) and Millipore AP prefilt (D = 150 mm, MERCK Millipore, Darmstadt, Germany). The solution was concentrated by ultrafiltration through an XM300 membrane (>300,000 MW; XM300 quantly 25, MERCK Millipore, Darmstadt, Germany) to cut off large molecules with a molecular weight above 300 kDa. Filtration mode: pressure was increased from 7 psi to 20 psi (0.5–1.5 atm) at a temperature of 25–30 °C. The retentate was purified by dialysis, washing five times with phosphate buffer (pH 7.1), and purification was controlled by measuring the optical density on a spectrophotometer (SF-2000, OKB Spectr, Saint-Petersburg, Russia) in the wavelength range 200–1000 μm with a step of 0.1 μm. The retentate was removed, and the permeate was passed through an XM150 membrane (150,000 MW; XM150 quantly 25, MERCK Millipore, Darmstadt, Germany) under the following conditions: filtration mode, pressure increased from 7 psi to 25 psi (0.5–2.0 atm.); temperature of 25–30 °C. The final purification step was with the UM20E membrane (>15–25,000 MW; UM20E quantly 25, MERCK Millipore, Darmstadt, Germany) under the following conditions: filtration mode, pressure increased from 15 psi to 75 psi (1.0–5.0 atm.); temperature of 25–30 °C. To isolate the homogeneous enzyme, high-performance liquid chromatography (HPLC NGC, BIO-RAD, Hercules, CA, USA) and the Sephadex G100 sorbent were used.

The activity of LO in the culture liquid of the producer *Trichoderma harzianum Rifai* was determined at reference values of 0.54–0.58 U/mg. The activity of LO in CL was determined in the range from 2.84 to 2.88 U/mg. For LO purification, high-performance liquid chromatography was used.

### 2.2. Microorganisms

The following strains of microorganisms were used in the study: *Enterococcus faecalis* ATTC 5960, *Enterococcus avium* 1669, *Enterococcus cloacae* 6392, *Klebsiella oxytoca* 3003, *Moraxella catarrhalis* 4222, *Morganella morganii* 1543, *Proteus mirabilis* 1543, *Pseudomonas aeruginosa* 3057, *Staphylococcus aureus* 4785, *Staphylococcus aureus* 6538, *Staphylococcus simulans* 5882, *Streptococcus hominis* 19, *Streptococcus agalacticae* 3984, Streptococcus mutans 21, and Candida spp. (*Candida albicans*, *Candida parapsilosis*, *Candida pelliculosa*, *Candida tropicalis*).

All the strains were isolated from patients of different genders aged from 2 to 17 years with a clinical diagnosis of urethritis/cystitis (80 patients); 63 of them had undergone bladder catheterization. Microbial strains were deposited in the microbiological bank at −70 °C in the Department of Microbiology named after. V. S. Kiktenko, Medical Institute, RUDN University, and used for this study.

The identification of microorganisms, bacteria, and fungi was carried out using the mass spectrometric method MALDI TOF MS on a Vitek MS Plus device (bioMérieux; Craponne France) and the Saramis Premium program v. 4.10 in accordance with the recommendations of the equipment manufacturer. Some of the microorganisms were identified using Sanger sequencing of the amplified fragments of the 16S rRNA gene region. First, the polymerase chain reaction (PCR) was used to amplify a region of the 16S rRNA gene using universal bacterial primers, 27F (5′-AGAGTTTGATCCTGGCTCAG-3′) and 1492R (5′-ACGGTACCTTGTTACGACTT-3′). The following program was used: 20 s denaturation at 94 °C; 20 s primer annealing at 58 °C; 90 s elongation at 72 °C, 40 cycles. The Cleanup Standard kit (Evrogen, Moscow, Russia) was used to purify the resulting amplification product. Sanger sequencing of the amplified DNA fragment from the UF1 primer was carried out at the Evrogen company (Moscow, Russia). Chromas Lite software, version 2.6.6 (Technelysium Pty. Ltd.; Australia), was used to visually determine the cutoff boundaries of the electropherogram sequences. The species of bacteria were determined based on a search for the obtained nucleotide sequences in the GenBank database using the Megablast algorithm. A comparison was considered to be at the species level if its partially sequenced 16S rRNA gene sequence had ≥98.7% similarity to the sequence of the closest known bacterial species in GenBank.

### 2.3. Antibiotic Resistance Tests

For each microorganism, sensitivity to the following antibiotics (HiMedia, Mumbai, India) was determined: Amoxiclav (AM)—30 µg/disk; Ampicillin (AMP)—25 µg/disk; Ceftazidime with clavulanic acid (CAC)—30/10 µg/disk; Ceftazidime (CAZ)—30 µg/disk; Ceftriaxone (CTR)—30 µg/disk; Imipenem (IMP)—10 mg/disk; Nitrofurantoin (NIT)—200 µg/disk; Trimethoprim (TR)—30 µg/disk; Ciprofloxacin (CIP)—30 µg/disk; Tetracycline (TE) 30 µg/disk.

The modified Kirby–Bauer well-diffusion method was used to study the antibacterial effect of LO in relation to the studied uropathogenic microorganisms [24,25]. For the cultivation of microorganisms, the following conditions were used: brain–heart broth (HiMedia, Mumbai, India), an incubation time of 24 h, and a temperature of 37 °C. After that, the broth cultures were pelleted by centrifugation under the following conditions—2.4 × 10^3^ rpm for 10 min (CM-6M centrifuge, ELMI SkyLine, Riga, Latvia). For the subsequent preparation of a suspension of microorganisms in a physiological solution (0.9% NaCl) at a concentration of 1.5–3.0 × 10^8^ CFU/mL, 0.5 turbidity standards (McFarland, HIMEDIA) were used. After incubation for 24 h at 37 °C, wells with a diameter of 5 mm and a volume of 15 ± 1 μL were made in agar using a sterile metal punch. LO was added to the wells at the following concentrations: 0.0001, 0.001, 0.01, 0.025, 0.05, 0.075, and 0.1 g/mL. A 0.9% sodium chloride solution and a fosfomycin test disk (FO200 (200 μg/disk) HiMedia, Mumbai, India) were used as the negative control and positive control, respectively.

The multidrug resistance index (MDRI) was calculated as the number of antibiotics to which the microorganism is resistant, divided by the total number of antibiotics in the experiment.

### 2.4. Biofilm Formation Ability

The microplate culture method was used to evaluate the inhibition of biofilm formation by LO/cCL. For the cultivation of microorganisms, the following conditions were used: brain–heart broth (HiMedia, Mumbai, India), an incubation time of 24 h, and a temperature of 37 °C. For the subsequent preparation of a suspension of microorganisms in a physiological solution (0.9% NaCl) at a concentration of 1.5–3.0 × 10^8^ CFU/mL, 0.5 turbidity standards (McFarland, HiMedia, Mumbai, India) and a densitometer (Biosan, DEN-1B, Riga, Latvia) were used. A sterile solution of 0.9% sodium chloride was added to the bacterial suspension in a ratio of 1/20. The prepared bacterial suspensions were used to inoculate the wells of sterile polystyrene 96-well plates. In each well, 20 μL of bacterial suspension and 180 μL of sterile liquid Mueller–Hinton medium with a volume of 180 μL were added. The final dilution of the suspensions was 1 to 10. Then, LO at a final concentration of 0.1 mg/mL and cCL with a final dilution of 1:10 were added to the appropriate holes. The additions of LO and cCL solutions were carried out in three variants: at the beginning of the experiment (0 h of cultivation), one day later (24 h of cultivation), and two days (48 h of cultivation) after the start of cultivation. The total time of cultivation of microorganism strains, regardless of the time of application of the test substances, was 72 h at 37 °C. After cultivation, the wells of the plate were washed three times with phosphate buffer (PBS) and dried for 15 min. Then, a 1% crystal violet solution was added to each well. The wells were incubated for 15 min, washed again with PBS solution, and dried for 15 min. After that, 150 μL of 96% ethanol was added to each well. Each well was incubated for 15 min, and the results were recorded on a microplate reader at a wavelength of 492 μm.

### 2.5. Scanning Electron Microscopy (SEM)

To identify changes in the morphology of the studied microorganisms during growth on a solid nutrient medium in the presence of a culture liquid concentrate and a homogeneous enzyme solution, the test substances were added in an amount of 10 mL to 90 mL (1:10) of molten Mueller–Hilton agar (HiMedia, Mumbai, India) (at a nutrient medium temperature of 43 °C to avoid the denaturation and inactivation of the enzyme) with a 1% glucose solution. Microporous nylon membranes with a pore diameter of 0.45 microns (Tecnofilter, Moscow, Russia) were placed on the surface of the solidified agar, followed by the application of a suspension of microorganisms. Biofilms were formed within 48 h. To conduct an electron microscopic examination, biological objects were fixed with 25% glutaraldehyde vapor for 24 h. Control samples were stabilized before the experiment. Pieces of membrane filters with biofilms were dehydrated twice with propylene oxide vapor. To remove excess charge from the surface of the sample, gold ions were sprayed onto the surface of the sample to create a conductive layer.

A morphological study of biofilms was carried out on a TESCAN Mira scanning electron microscope (Tescan, Brno, Czech Republic) using reflected and secondary electron (SE) detectors. The accelerating voltage was 7 kV, and the beam current was 2.5–7 nA. This gentle method of sample preparation and research allowed us to identify the natural structural organization of the microbial biofilm.

### 2.6. Statistical Analysis

Experiments were carried out in ten (disco-diffusion method) and twenty biological replicates. The results are presented as means ± standard deviations. Spearman’s method was used to analyze correlation dependence. The Programs XLSTAT (Premium v2016.02.28451) and RStudio v. 1.6.0. (R ver. 4.3.2.) were used to process the obtained data.

## 3. Results

### 3.1. Antibiotic Resistance Tests

The following research results were obtained: LO has a more pronounced antibacterial effect against Gram-positive bacteria and a less pronounced effect against Gram-negative bacteria (Table 1).

Additionally, for each microorganism, the antibiogram was determined (Table 2), and the multidrug resistance index (MDRI) was calculated; then, the microorganisms were listed. In the following, the antibiotics in brackets are those to which resistance was identified, and the MDRI values are indicated: *Enterococcus faecalis* 5960 (TE, CAC, AMP, TR; MDRI = 0.364), *Enterococcus avium* 1669 (TE, CAC, AMP, TR; MDRI = 0.364), *Enterococcus cloacae* 6392 (NIT, TE, CAC, AMP, TR; MDRI = 0.455), *Klebsiella oxytoca* 3003 (TE, AMP, CAZ, CAC, TR; MDRI = 0.364), *Moraxella catarrhalis* 4222 (NIT, CAZ, AMP, TR; MDRI = 0.364), *Morganella morganii* 1543 (NIT, TE, FO, AMP, TR; MDRI = 0.455), *Proteus mirabilis* 1543 (TE, AMP, TR; MDRI = 0.273), *Pseudomonas aeruginosa* 3057 (NIT, TE, CAZ, AMP, TR; MDRI = 0.455), *Staphylococcus aureus* 4785 (NIT, CAZ, CAC, AMP, TR; MDRI = 0.455), *Staphylococcus aureus* ATTC 6538 (MDRI = 0.000), *Staphylococcus simulans* 5882 (CAZ, CAC, AMP; MDRI = 0.273), *Streptococcus hominis* 19 (TE; MDRI = 0.182), *Streptococcus agalacticae* 3984 (NIT, TE, CAZ, CAC, AMP, TR; MDRI = 0.545), *Streptococcus mutans* 21 (TE, AMP; MDRI = 0.273).

### 3.2. Biofilm Formation

**Figure 1:**
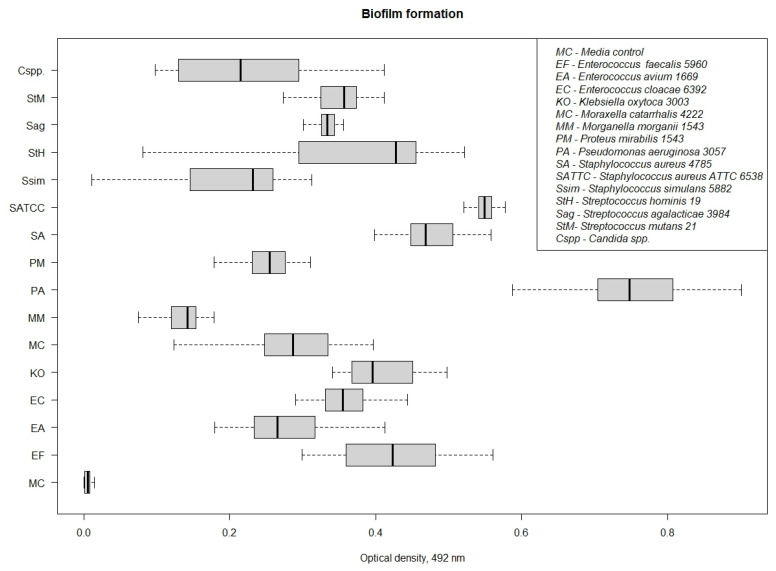
Level of biofilm formation in microorganisms associated with urethritis/cystitis during microplate cultivation.

After 72 h of cultivation on a microplate, washing and staining of the biofilm, additional washing, and the extraction of dye from the formed biofilm by adding 96% ethanol, the optical density of each well was recorded on a plate reader at a length of 492 μm. The results are presented in Figure 1.

As can be seen from the data obtained, the most effective (>0.5, at a wavelength of 492 μm) formation of biofilms was observed for such microorganisms as *Pseudomonas aeruginosa* 3057 (M = 0.739 ± 0.135) and *Staphylococcus aureus* 6538 (M = 0.551 ± 0.015). The microorganisms *Enterococcus faecalis* 5960 (M = 0.421 ± 0.069), *Enterococcus avium* 1669 (M = 0.278 ± 0.060), *Enterococcus cloacae* 6392 (M = 0.359 ± 0.038), *Klebsiella oxytoca* 3003 (M = 0.426 ± 0.1 69), *Moraxella catarrhalis* 4222 (M = 0.290 ± 0.079), *Morganella morganii* 1543 (M = 0.140 ± 0.033), *Proteus mirabilis* 1543 (M = 0.251 ± 0.031), *Staphylococcus aureus* 4785 (M = 0.479 ± 0.039), *Staphylococcus simulans* 5882 (M = 0.210 ± 0.087), *Streptococcus hominis* 19 (M = 0.365 ± 0.139), *Streptococcus agalacticae* 3984 (M = 0.329 ± 0.055), *Streptococcus mutans* 21 (M = 0.352 ± 0.038), and *Candida* spp. (M = 0.219 ± 0.089) showed less pronounced results, but based on the results of the experiment, we can say that they were able to form biofilms.

#### Suppression of Biofilm Formation

We analyzed the formation of biofilms by the studied isolates of microorganisms using the microplate cultivation method in Mueller–Hinton broth after 72 h of cultivation with the addition of the homogeneous enzyme or culture liquid at different stages of biofilm formation using the microplate cultivation method. *Candida albicans*, *Candida parapsilosis*, *Candida pelliculosa*, and *Candida tropicalis* showed no inhibition of biofilm formation, so for convenience, they are combined into *Candida* spp.

**Figure 2:**
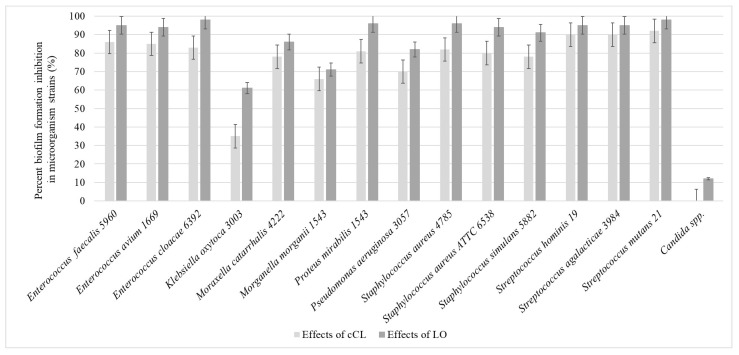
The efficiency of suppression of biofilm formation when adding *T. harzianum Rifai* cCL and LO to the nutrient medium at the beginning of the experiment (*p* < 0.05).

According to the recorded results, it was revealed that when substances are added at the beginning of the experiment, LO caused the pronounced suppression of biofilms in relation to all microorganisms that were tested in the experiment, and cCL, although it had a less pronounced effect, still suppressed the process of biofilm formation by more than 50%: *Enterococcus faecalis* 5960—cCL 86%, LO 95%; *Enterococcus avium* 1669—cCL 85%, LO 94%; *Enterococcus cloacae* 6392—cCL 83%, LO—98%; *Moraxella catarrhalis* 4222—cCL 78%, LO—86%; *Morganella morganii* 1543—cCL 66%, LO—71%; *Proteus mirabilis* 1543—cCL 81%, LO—96%; *Pseudomonas aeruginosa* 3057—cCL 70%, LO—82%; *Staphylococcus aureus* 4785—cCL 82%, LO—96%; *Staphylococcus aureus* 6538—cCL 80%, LO—94%; *Staphylococcus simulans* 5882—cCL 78%, LO—91%; *Streptococcus hominis* 19—cCL 90%, LO—95%; *Streptococcus agalacticae* 3984—cCL 90%, LO—95%; *Streptococcus mutans* 21—cCL 92%, LO—98%. The effect on the *Klebsiella oxytoca* 3003 strain was significantly smaller—cCL 35%, LO—61%. There was no significant effect on the yeast-like fungi *Candida* spp.: when cCL was added, no suppression was observed, and when LO was added, the percentage of suppression was only 12% on average (Figure 2).

**Figure 3:**
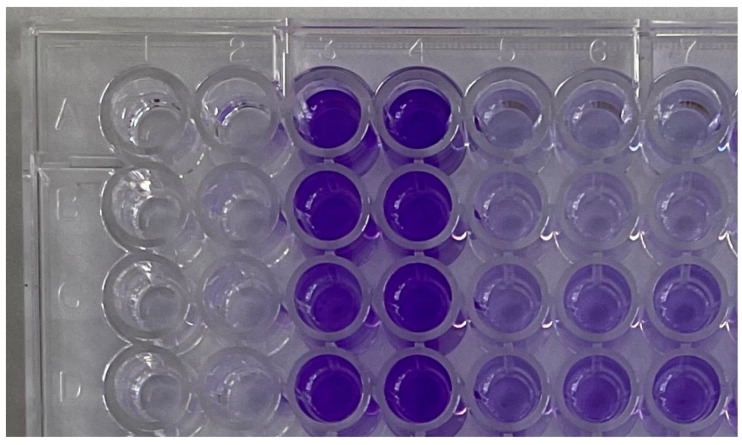
Tablet method for determining biofilm formation in *Staphylococcus aureus* ATCC 6538 strain and suppressing biofilm formation by adding enzyme L-lysine-Alpha-oxidase. Wells: A1–D2—medium control; wells A3–D4—culture control; wells A5–D 7—experimental wells with a microorganism culture and the added enzyme L-lysine-α-oxidase at a final concentration of 1 mg/mL.

This level of suppression of biofilm formation was observed only when the cCL or LO solution was added at the early stages of biofilm formation (up to 24 h of cultivation). At later stages—24 and 48 h of cultivation—the studied samples either suppressed the formation by several times less or no longer had any effect on the process of biofilm formation (Figure 3).

**Figure 4:**
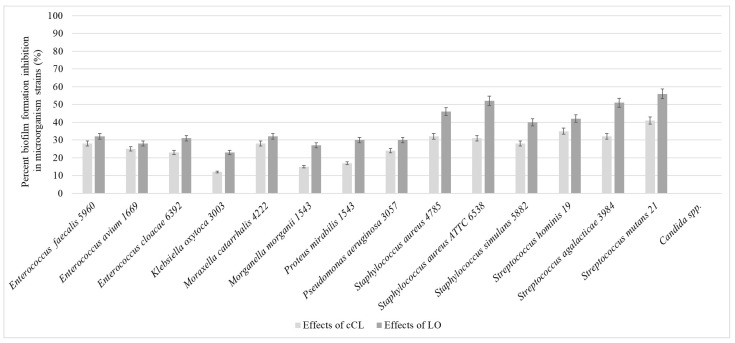
The efficiency of suppression of biofilm formation when introducing *T. harzianum Rifai* cCL and LO into the nutrient medium at the stage of 24 h culture cultivation (*p* < 0.05).

When the solutions were added after 24 h of cultivation, the following results were obtained: *Enterococcus faecalis* 5960—cCL 28, LO—32%; *Enterococcus avium* 1669—cCL 25%, LO—28%; *Enterococcus cloacae* 6392—cCL 23%, LO—31%; *Klebsiella oxytoca* 3003—cCL 12%, LO—23%; *Moraxella catarrhalis* 4222—cCL 28%, LO—32%; *Morganella morganii* 1543—cCL 15%, LO—27%; *Proteus mirabilis* 1543—cCL 17%, LO—30%; *Pseudomonas aeruginosa* 3057—cCL 24%, LO—30%; *Staphylococcus aureus* 4785—cCL 32%, LO—46%; *Staphylococcus aureus* 6538—cCL 31%, LO—52%; *Staphylococcus simulans* 5882—cCL 28%, LO 40%; *Streptococcus hominis* 19—cCL 35%, LO 42%; *Streptococcus agalacticae* 3984—cCL 32%, LO—51%; *Streptococcus mutans* 21—cCL 41%, LO—56%; *Candida* spp.—cCL and LO 0% (Figure 4).

**Figure 5:**
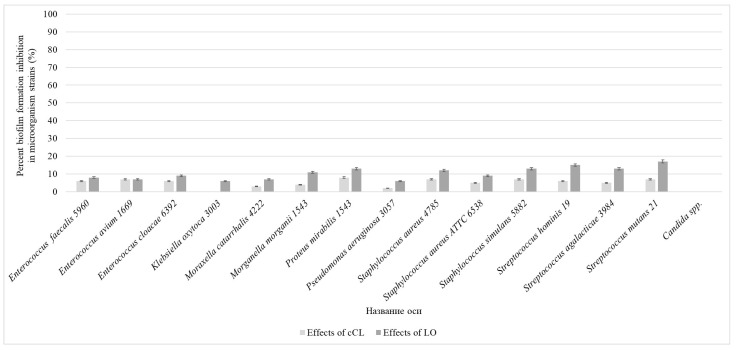
The efficiency of suppression of biofilm formation when adding *T. harzianum Rifai* cCL and LO to the nutrient medium at the stage of 48 h culture cultivation (*p* < 0.05).

When adding cCL *T. harzianum Rifai* and LO to the nutrient medium after 48 h of cultivation of microorganisms, the following results were obtained: *Enterococcus faecalis* 5960—cCL 6%, LO 8%; *Enterococcus avium* 1669—cCL 7%, LO 7%; *Enterococcus cloacae* 6392—cCL 6%, LO 9%; *Klebsiella oxytoca* 3003—cCL 0%, LO 6%; *Moraxella catarrhalis* 4222—cCL 3%, LO 7%; *Morganella morganii* 1543—cCL 4%, LO 11%; *Proteus mirabilis* 1543—cCL 8%, LO 13%; *Pseudomonas aeruginosa* 3057—cCL 2%, LO 6%; *Staphylococcus aureus* 4785—cCL 7%, LO 12%; *Staphylococcus aureus* 6538—cCL 5%, LO 9%; *Staphylococcus simulans* 5882—cCL 7%, LO 13%; *Streptococcus hominis* 19—cCL 6%, LO 15%; *Streptococcus agalacticae* 3984—cCL 5%, LO 13%; *Streptococcus mutans* 21—cCL 7%, LO 17%; *Candida* spp.—cCL and LO—0% (Figure 5).

### 3.3. Scanning Electron Microscopy (SEM)

The effect on the process of biofilm formation was studied by introducing the test substances to nutrient media. Scanning electron microscopy revealed the morphological features of the microorganisms.

**Figure 6:**
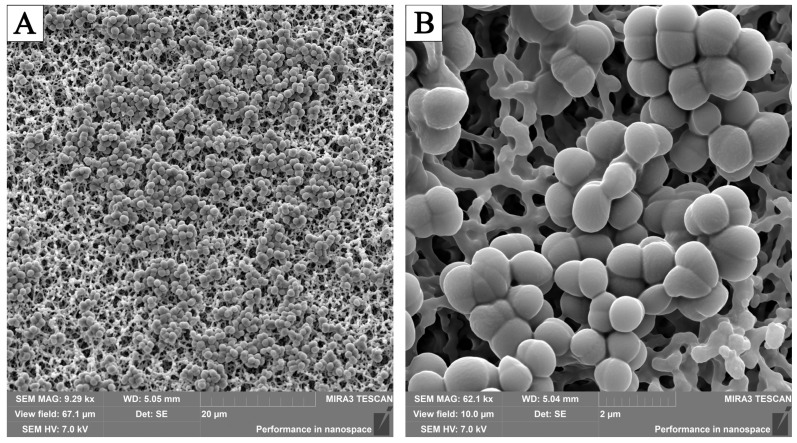
Single microcolonies of *Staphylococcus aureus* 4785 in a zone with no visible growth when cCL was added to the nutrient medium ((**A**)—magnification 9.29 kx; (**B**)—magnification 62.1 kx). SEM.

When LO was added to the nutrient media in areas without visible growth of *staphylococci*, single clusters of polymorphic cells of different sizes were noted (Figure 6).

**Figure 7:**
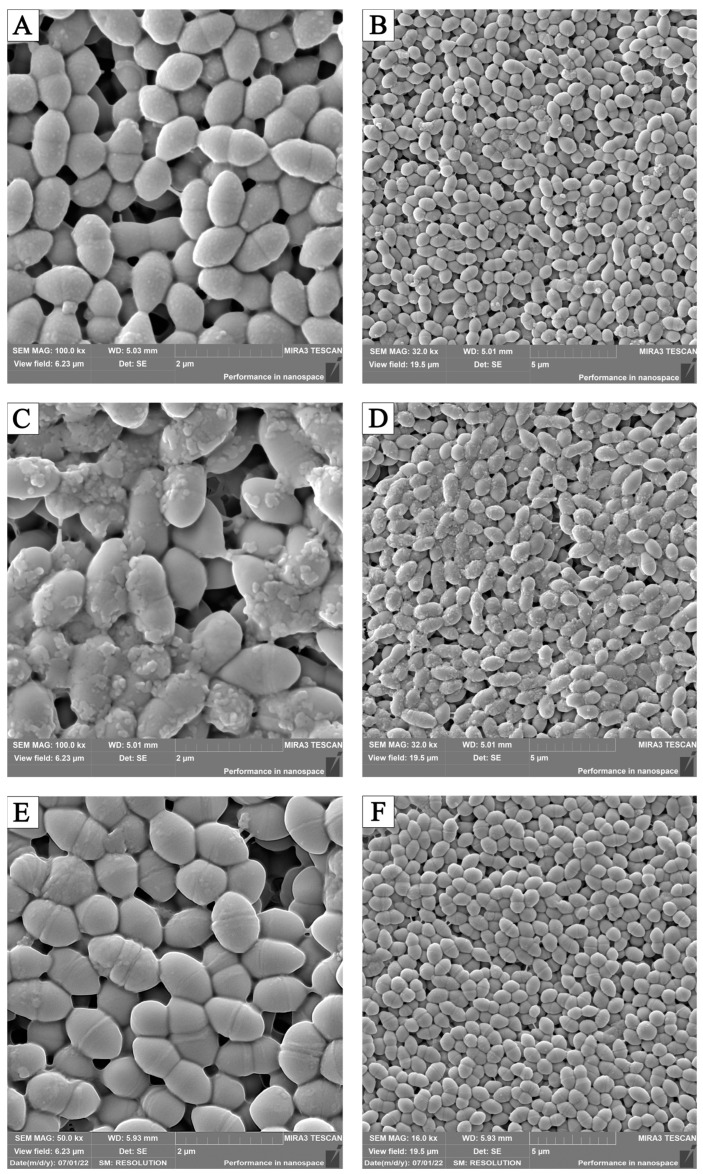
Morphology of *Enterococcus faecalis* 5960: (**A**,**B**)—controls; images of cells after adding the following to the nutrient medium: (**C**,**D**)—cCL of *Trichoderma Harzianum Rifai*; (**E**,**F**)—LO. SEM.

The addition of the culture liquid to the nutrient medium led to the appearance of flocculent polymorphic masses of *Enterococcus faecalis* 5960 on the surface, while after exposure to the enzyme, cases of abnormal division were detected, which is manifested by the appearance of joints at the places where the bacteria diverge (Figure 7). In several cases, the appearance of a dense film of intercellular matrix covering the microbial population was noted. It was established that in areas of thin biofilms, along with the absence of signs of cell division, there were threads/strands that united round-shaped bacteria lying on the surface of the membrane filter. In the deep layers of the biofilms, part of the population was round; the bacteria were connected in pairs by dense formations that were longer than the total diameter of the combined bacteria.

**Figure 8:**
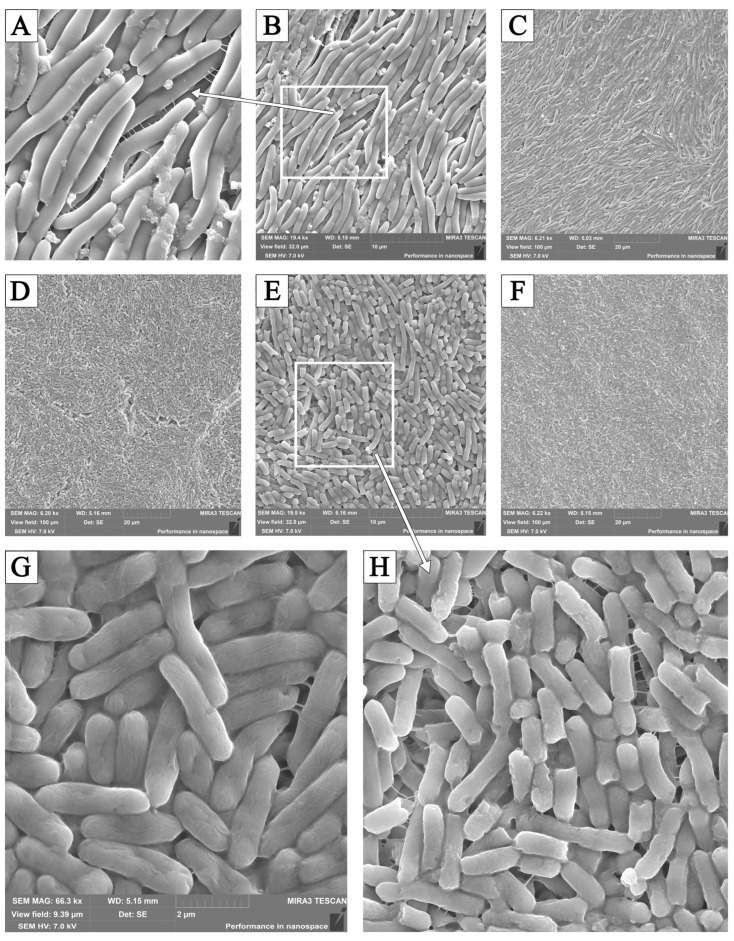
Biofilm fragments of *Pseudomonas aeruginosa* 3057 (**A**–**C**)—controls; after exposure to: (**D**,**G**)—cCL; (**E**,**F**)—LO; (**H**)—enlarged fragments. SEM.

It was found that, in control samples, *Pseudomonas aeruginosa* 3057 formed long chains of bacteria united by a common capsule, under which the outlines of bacteria were clearly visible. When growing in the presence of cCL, the population was represented by smaller rods with chopped-off/blunt ends, and defects of various shapes and depths were noted. Adding LO to the nutrient medium deprived the bacteria of the unifying capsule; flagella were visible on the surface of clearly visible bacteria (Figure 8).

## 4. Discussion

The mechanism of the suppression of biofilm formation with the addition of LO and cCL is possibly based on the competitive binding of lysine to the enzyme, resulting in the disruption of the synthesis of the intercellular matrix, where this amino acid is involved. On the other hand, the reception of signaling molecules may be impaired, for example, in proteins of the QS family, the synthesis of which may be significantly reduced due to a lack of substrate.

These assumptions were confirmed by the results of electron microscopy. Multiple defects in the cell wall could be observed under the influence of LO and cCL, which appeared as a result of the inhibition of the synthesis of necessary structural proteins and an atypical arrangement of cells compared to control samples, which may indicate, on the one hand, a decrease in or the absence of the synthesis of adhesion proteins and, on the other hand, the absence of or change in the production of molecules responsible for “intercellular communication”.

For some microorganisms, for example, representatives of the *Enterobacteriaceae* and *Pseudomonadaceae* families, the amino acid lysine is involved in protein malonylation [26], and destruction by the L-lysine enzyme probably leads to the disruption of protein translation and folding, which will certainly lead to the inhibition of biofilm formation. In addition, there is probably a similar mechanism of inhibition of the synthesis and activity of enzymes involved in the processes of capsule synthesis, which we can observe in electron micrographs. In our study, the most pronounced effect was on *Pseudomonas aeruginosa*, the samples of which showed no capsule formation under electron microscopy, in contrast to the control. Theoretically, the absence of a capsule may be due to the dehydration of the polysaccharide residues that make up the capsule. This process can theoretically occur under the influence of carbolic acid formed as a reaction product during the interaction of the enzyme LO with lysine. The degree of the degradation of the capsule, as well as the inhibition of its synthesis, is probably related to the polysaccharide composition of the structure and varies depending on the type of microorganism.

It is possible that, in addition to the above, the enzyme disrupts the primary adhesion of bacterial cells, again due to the disruption of the synthesis of adhesins and capsules due to a decrease in the amount of lysine. It is most likely that these factors interact together.

An interesting observation is the minimal decrease in biofilm formation by representatives of the genus *Candida* [9]. There are probably other signaling molecules and a different matrix composition, and the enzyme may have difficulty penetrating the fungal cell, which has a more rigid cell wall. Moreover, in representatives of the genus *Candida*, during the formation of biofilms, processes that differ from those observed for bacteria are described; for example, during adhesion, these fungi form microtubules and synthesize many enzymes for the penetration and destruction of the cell walls of eukaryotes [27,28,29]. It is theoretically possible that these aggressive *Candida* enzymes can destroy or inactivate the enzyme L-lysine-α-oxidase or that the chitinous layer is too tough for LO.

A difference between the effects of LO and cCL was demonstrated: the homogeneous enzyme was always more effective in inhibiting biofilms than the cCL culture fluid concentrate. This was probably due to the lower concentration of the enzyme itself in the culture fluid concentrate, but this allowed us to conclude that it is the enzyme L-lysine-α-oxidase that suppresses the process of biofilm formation and not any other metabolic product of the producing fungus *Trichoderma harzianum Rifai*.

According to other studies, the enzyme has low allergenicity and immunogenicity [24], which, together with the ability to inhibit the formation of biofilms by microorganisms, makes this substance a worthy candidate for coating medical devices, such as a urinary catheter. Theoretically, such a coating will reduce the risk of infection and/or prevent the formation of biofilms by pathogenic bacteria causing infections of opportunistic and nosocomial origins; that is, it will reduce possible complications of treatment.

In this study, sensitivity to antibiotics was determined for each strain, and the MDRI was calculated: the highest index was for *Streptococcus agalacticae* 3984 (NIT, TE, CAZ, CAC, AMP, TR; MDRI = 0.545), and the lowest for *Staphylococcus aureus* 6538 (MDRI = 0.000).

As a result of the study, it was revealed that all studied strains can form biofilms; the highest abilities were shown by *Pseudomonas aeruginosa* 3057 (M = 0.739 ± 0.135) and *Staphylococcus aureus* 6538 (M = 0.551 ± 0.015), and the lowest by *Streptococcus mutans* 21 (M = 0.352 ± 0.038) and *Candida* spp. (M = 0.219 ± 0.089). A comprehensive study of biofilm formation was carried out, and the morphological characteristics of biofilms of microorganisms of various taxonomic groups during growth in the presence of the studied substances were studied.

The greatest effect was recorded when adding LO and cCL at 0 h of incubation and when adding LO to *Enterococcus faecalis* and *Enterococcus avium* 1669, with decreases of 95 and 94%, respectively, and the smallest when adding cCL to *Candida* spp.—0%—with a 12% decrease when adding LO to this culture.

When LO and cCL were added at 24 h of cultivation, the inhibition efficiency decreased significantly, for most cultures by more than two times. When LO and cCL were added, almost no suppression of biofilm formation was observed. At the same time, as the number of incubation hours before adding LO and cCL increases, the efficiency decreases, so we can conclude that LO and cCL suppress the formation of biofilms but do not significantly affect those that have already formed. There are significant morphological changes in the bacterial elements of the biofilm when formed on a medium with the addition of cCL and LO. In electron micrographs, the following were observed in cultures sensitive to the enzyme: numerous cell wall defects, a lack of capsule synthesis, atypically located cells, and atypical cell division.

## 5. Conclusions

The purpose of this study was to study the effect of the homogeneous enzyme L-lysine-α-oxidase produced by the fungus *Trichoderma harzianum Rifai* on the process of biofilm formation by microorganisms associated with urinary tract infections. LO showed a more pronounced effect than the culture liquid concentrate (cCL). When adding samples at the beginning of incubation, maximum inhibition was observed in relation to *Enterococcus faecalis* 5960—cCL 86%, LO 95%; *Enterococcus avium* 1669—cCL 85%, LO 94%; *Enterococcus cloacae* 6392—cCL 83%, LO—98%; and *Pseudomonas aeruginosa* 3057—cCL 70%, LO—82%. The minimum inhibition was observed in *Candida* spp. Scanning electron microscopy revealed numerous morphological and structural changes in cells after culturing bacterial cultures in a medium supplemented with the homogeneous LO enzyme. Based on the results of this work, we can make conclusions about the possibility of inhibiting biofilm formation in most of the studied microbial cultures using the enzyme L-lysine-α-oxidase, but significant inhibition was achieved only when the enzyme was added at the beginning of incubation.

However, the speculated mechanism of action for LO/cCL is the competitive binding of lysine to the enzyme and the disruption of matrix formation. This should be tested further. An experiment can be designed in which exogenous lysine is added to the media at the start of the experiment at the same time as the enzyme. If it is competitive binding, then adding extra lysine would lead to less biofilm inhibition. This can be performed with the two strains affected the least by toxicity from the enzyme, *Enterococcus cloacae* 6392 and *Pseudomonas aeruginosa* 3057.

The potential application of LO as a coating for medical devices is a promising avenue. However, further discussion on the practical implications, challenges, and potential risks associated with introducing this enzyme into clinical settings is warranted. Addressing concerns related to safety and compatibility with human tissues is crucial.

## Figures and Tables

**Table 1:** Fixed zones of growth inhibition by test microorganisms when adding LO to wells (mm).

Dependence of Efficiency on Concentration of Homogeneous Enzyme in Solution							
Microorganism, Strain	Enzyme Concentration (50 U/mg) in g/mL Solution						
	0.1	0.075	0.05	0.025	0.01	0.001	0.0001
*Enterococcus faecalis* 5960	14 ± 0.44	12 ± 0.45	10 ± 0.75	8 ± 0.44	6 ± 0.77	0	0
*Enterococcus avium* 1669	14 ± 0.51	14 ± 0.44	12 ± 0.89	10 ± 0.83	7 ± 0.89	0	0
*Enterococcus cloacae 6392*	12 ± 0.54	11 ± 0.63	11 ± 1.14	8 ± 0.89	0	0	0
*Klebsiella oxytoca* 3003	13 ± 0.7	12 ± 0.83	10 ± 1.14	7 ± 0.83	6 ± 0.89	0	0
*Moraxella catarrhalis* 4222	12 ± 0.89	11 ±1.14	9 ± 0.44	7 ± 0.75	6 ± 0.54	0	0
*Morganella morganii* 1543	13 ± 0.45	12 ± 0.71	10 ± 0.83	7 ± 0.89	6 ± 0.45	0	0
*Proteus mirabilis* 1543	14 ± 0.71	13 ± 0.44	11 ± 1.0	8 ± 0.84	6 ± 0.89	0	0
*Pseudomonas aeruginosa* 3057	11 ± 0.77	10 ± 0.54	8 ± 1.0	6 ± 0.89	0	0	0
*Staphylococcus aureus* 4785	21 ± 1.3	19 ± 1.14	16 ± 0.44	13 ± 0.75	12 ± 0.63	7 ± 0.41	0
*Staphylococcus aureus ATTC* 6538	20 ± 0.75	18 ± 0.54	14 ± 0.89	12 ± 0.63	10 ± 0.83	6 ± 0.49	0
*Staphylococcus simulans* 5882	21 ± 0.51	18 ± 0.54	16 ± 0.83	13 ± 0.63	9 ± 0.37	7 ± 0.44	0
*Streptococcus hominis* 19	18 ± 0.44	16 ± 0.83	13 ± 0.77	12 ± 0.54	12 ± 0.63	6 ± 0.45	0
*Streptococcus agalacticae* 3984	18 ± 0.71	15 ± 0.89	13 ± 0.84	12 ± 0.54	10 ± 0.77	8 ± 0.44	0
*Streptococcus mutans* 21	23 ± 0.77	19 ± 0.71	14 ± 0.54	13 ± 0.44	13 ± 1.14	7 ± 0.45	0

**Table 2:** Fixed zones of growth inhibition in Kirby–Bauer test (mm).

Microorganism Strain	Antibiotics										
	NIT	TE	CTR	AMC	FO	CAZ	IPM	CAC	CIP	AMP	TR
*Enterococcus faecalis* 5960	22 ± 1.03	0	20.5 ± 1.04	28.5 ± 0.54	31 ± 0.89	25 ± 0.51	26.5 ± 0.81	0	24 ± 0.75	0	0
*Enterococcus avium* 1669	21 ± 0.63	6 ± 1.18	23 ± 0.41	30 ± 0.51	32.5 ± 0.82	28 ± 0.75	25 ± 0.51	0	26 ± 0.63	0	0
*Enterococcus cloacae* 6392	12.5 ± 0.83	11 ± 0.51	22 ± 0.82	28 ± 0.89	26 ± 0.52	22.5 ± 1.2	28.5 ± 0.82	0	22 ± 0.62	0	7 ± 0.53
*Klebsiella oxytoca* 3003	19 ± 0.63	0	21 ± 0.78	26.5 ± 0.82	20 ± 0.78	12.5 ± 0.54	27 ± 1.2	10 ± 1.27	29 ± 0.83	0	0
*Moraxella catarrhalis* 4222	6 ± 0.74	21 ± 0.75	24.5 ± 0.98	21.5 ± 0.81	24.5 ± 0.54	8 ± 0.75	20 ± 0.89	18 ± 0.75	22.5 ± 1.04	0	12 ± 1.39
*Morganella morganii* 1543	10 ± 0.51	0	19 ± 0.89	25 ± 1.51	31 ± 0.84	21 ± 0.95	27 ± 1.1	20.5 ± 1.21	18 ± 0.54	0	0
*Proteus mirabilis* 1543	31 ± 1.47	0	20 ± 1.03	28 ± 0.75	22 ± 0.51	24 ± 0.54	28 ± 0.75	19 ± 1.36	29 ± 0.54	8 ± 1.06	0
*Pseudomonas aeruginosa* 3057	0	0	25 ± 0.41	32 ± 1.51	28 ± 0.54	12 ± 0.41	25 ± 1.37	23 ± 1.21	34.5 ± 1.47	0	0
*Staphylococcus aureus* 4785	13 ± 0.84	26 ± 1.51	21 ± 0.81	27 ± 0.41	29 ± 0.54	0	24 ± 0.51	0	20 ± 0.41	0	0
*Staphylococcus aureus ATTC* 6538	25 ± 0.41	23 ± 0.89	19 ± 1.47	28 ± 1.96	27 ± 0.54	19 ± 0.98	24 ± 1.37	22 ± 0.63	20 ± 0.75	19 ± 0.75	21 ± 0.84
*Staphylococcus simulans* 5882	21 ± 0.81	19 ± 1.6	23 ± 0.41	30 ± 0.83	28 ± 0.89	8 ± 0.9	26 ± 0.81	6 ± 0.48	22 ± 0.63	8 ± 0.75	18 ± 0.54
*Streptococcus hominis* 19	27 ± 0.54	6 ± 0.53	23 ± 0.89	32 ± 1.3	30 ± 0.83	24 ± 0.71	23 ± 0.47	17 ± 0.98	26 ± 0.53	17 ± 0.81	24 ± 0.58
*Streptococcus agalacticae* 3984	11 ± 1.0	6 ± 0.98	25 ± 1.6	29 ± 0.51	31 ± 0.41	6 ± 0.48	27 ± 0.75	6 ± 0.74	28 ± 1.26	6 ± 0.98	7 ± 0.51
*Streptococcus mutans* 21	16 ± 0.51	7 ± 0.83	20 ± 1.16	25 ± 1.72	26 ± 1.32	16 ± 0.4	22 ± 0.75	19 ± 1.7	25 ± 0.63	8 ± 0.71	17 ± 0.54

## Data Availability

Upon request, the data will be made available from the corresponding author.
